# Atraumatic Gluteal Compartment Syndrome Presenting in a Young Female with Unilateral Lower Extremity Symptoms

**DOI:** 10.1155/2019/7891275

**Published:** 2019-01-29

**Authors:** Morgan Maxon, Curt Cackovic

**Affiliations:** Inspira Medical Center, Vineland, NJ 08360, USA

## Abstract

Gluteal compartment syndrome is a rare condition that often develops following immobilization either secondary to illicit drug and alcohol abuse or improper surgical positioning. A case of a 22-year-old female with left lower extremity pain, weakness, and numbness after prolonged stasis from a night of drug and alcohol use is presented. She also complained of left low back pain. Her initial neurologic examination was significant for decreased deep tendon reflexes, decreased motor strength, and decreased sensation in the left lower extremity. Severe pain in the affected region persisted despite several attempts at pain control utilizing multiple modalities. An emergent MRI of the lumbar spine revealed gluteal compartment syndrome. The patient ultimately underwent emergent fasciotomy with resultant improvement in neurologic symptoms. Because presenting symptoms are frequently nonspecific in initial stages, gluteal compartment syndrome is often misdiagnosed. This can lead to unnecessary morbidity and mortality. It is important to maintain a high index of suspicion for gluteal compartment syndrome because delay in diagnosis can lead to nerve palsy, acute kidney injury, sepsis, and/or death.

## 1. Introduction

Acute compartment syndrome is caused by fractures in the majority of cases. It most commonly occurs in the lower leg; the second most common site is the forearm.

Gluteal compartment syndrome is a very rare condition that may occur due to prolonged stasis. The very first case was described by Petrik in 1988. Only three nontraumatic cases have been reported in the literature. All three were either related to the use of heparin or antiplatelet medications [1]. Because symptoms are nonspecific, gluteal compartment syndrome is often misdiagnosed and can lead to unnecessary morbidity and mortality. Delay in diagnosis can lead to nerve palsy, acute kidney injury, sepsis, and/or death.

Diagnosis is generally made clinically; however, CT and MRI can help confirm the diagnosis [2]. In nearly all cases fasciotomy is the only definitive treatment. The pathophysiology, diagnosis, and treatment of gluteal compartment syndrome will be further explained.

## 2. Case Presentation

A 22-year-old female presented to the emergency department in a wheelchair with left lower extremity weakness and numbness, left low back pain, and left hip pain radiating to the left foot. She apparently had been found lying on the sidewalk. She stated that the pain felt similar to a “spasm”. There were no aggravating or alleviating factors. She had no similar prior episodes. She admitted to drinking alcohol and using illicit drugs the previous night. She reported that she awoke with the pain but could not remember if she had experienced a loss of consciousness. There was a large ecchymotic area on her left forehead, so she assumed that she had fallen. She could not remember any additional details. She noted that her leg pain has gotten progressively worse throughout the day.

Review of systems revealed ecchymosis to the left frontal area, pain in left lower extremity and hip with light palpation, decreased range of motion in left hip and knee, numbness of the left lower extremity, and weakness of the left lower extremity. Review of systems was negative for fever, chills, headache, chest pain, shortness of breath, nausea, vomiting, or change in urination or bowel movements. She had no known medical problems other than anxiety. She had no known drug allergies or previous surgeries. She endorsed illicit substance use and alcohol use.

Physical exam revealed a heart rate of 112 bpm, blood pressure of 145/95 mm Hg, respiratory rate of 18/ min, oral temperature of 98.1 degrees Fahrenheit, and oxygen saturation of 98% on room air. Her height was recorded as 5 feet 4 inches (162.6 cm), weight 143 pounds (64.9 kg), and body mass index (BMI) 24.5. The patient was in moderate distress, very uncomfortable, and restless. Her pupils were equal and reactive bilaterally. Extraocular muscles were intact. There were no signs of a basilar skull fracture. Nares were patent bilaterally. Hearing was normal. Tympanic membranes were clear. There was moderate sized ecchymosis to the left frontal region without any appreciable step-off or deformity. There was no bony midline cervical tenderness to palpation. Trachea was midline. Chest was nontender without deformity or crepitus. Heart was regular rate and rhythm. Lungs were clear to auscultation bilaterally with good excursion. Abdomen was nontender, nondistended, with normal bowel sounds in all four quadrants. Normal perfusion was exhibited in all extremities with brisk capillary refill of less than two seconds. She had 2+ pulses bilaterally (radial, femoral, and dorsalis pedis). Her left lower thoracic and lumbar regions were tender to palpation with associated decreased range of motion in all directions. Muscle strength was 5/5 in the upper extremities bilaterally. Muscle strength was 5/5 in the right lower extremity and 3/5 in the left lower extremity. Deep tendon reflexes were 1+ left Achilles, 1+ left patellar, 2+ right Achilles, 2+ right patellar, 2+ bilateral biceps, and 2+ bilateral triceps. Patient was extremely tender on palpation of her entire left lower extremity extending from the hip distally to her left foot. Left lower extremity range of motion was severely limited due to pain. Cranial nerves II-XII were intact bilaterally. She had an antalgic gait. Cerebellum testing was within normal limits. She had diminished sensation to light touch in her left lower extremity. Romberg testing was normal.

Test results revealed a serum alcohol level <10, a negative urine pregnancy test, and a serum creatine kinase (CK) level within normal limits. Basic laboratory evaluation including complete blood count, chemistry panel, and liver function testing was unremarkable. CT brain, CT cervical spine, X-rays of the thoracic spine, X-rays of the lumbar spine, and X-rays of the left hip were all negative.

The patient complained of persistent, severe pain despite multiple attempts at controlling her pain using different modalities. The on-call neurologist was consulted and recommended an emergent MRI of her lumbar spine. Patient was transferred from the ED to a tertiary care center where she underwent this study. MRI results ultimately revealed high intensity signal at the left quadratus femoris, gluteus maximus/medius/minimus, and piriformis with significant overlying subcutaneous edema and inflammation consistent with gluteal compartment syndrome. Patient subsequently underwent a fasciotomy with resultant improvement in her abnormal sensation and deep tendon reflexes.

## 3. Discussion

Gluteal compartment syndrome is a rare condition. Overall incidence is between 3-11% of cases of compartment syndrome. It occurs when tissue pressure exceeds perfusion pressure in the gluteal compartment, leading to necrosis [3]. It may develop after immobilization secondary to drug or alcohol abuse or improper surgical positioning. Uncommonly it can be caused by either anticoagulation or antiplatelet medications, respectively.

Compartment syndrome results from increased pressure in any muscle compartment large enough to decrease perfusion. The two most common causes are trauma and vascular disorders. Cellular anoxia caused by a reduced arteriovenous pressure gradient is the most commonly accepted pathophysiology [4]. This reduced gradient can cause nerve and muscle damage if not promptly identified. Decreased venous drainage leads to an increase in pressure, resulting in a rise in compartment pressure. Once the pressure rises within 10-30 mm Hg of diastolic pressure, oxygenation of muscle decreases. This sequence of events leads to cellular anoxia. Nerve conduction also decreases when pressure is increased; the more the pressure increases, the greater the likelihood that paralysis can occur. If pressure remains elevated for more than four hours, it can become irreversible. Since the gluteus medius muscle is smaller in size compared to gluteus maximus, compartment syndrome generally occurs in the medius more frequently.

On presentation, patients can have nonspecific symptoms such as swelling, pain, numbness, and weakness. Patients may also have a foot drop due to sciatic nerve and peroneal nerve involvement, respectively. Gluteal compartment syndrome is usually a clinical diagnosis; however, to differentiate from other causes of pain and swelling, labs and imaging may assist in making the diagnosis. CK level is often elevated. Compartmental pressures can also be measured to help determine the most likely cause of symptoms. Pressures greater than 30 mm Hg are common [5]. Imaging modalities such as CT, MRI, and ultrasound may be utilized. If late enough in the physiologic process, CK level and serum potassium will both be elevated. The patient's electrocardiogram may have dynamic changes secondary to hyperkalemia including peaked T waves and a widened QRS interval [6].

At approximately 4-8 hours after injury, damage becomes irreversible [7]. At this point, emergent fasciotomy is the gold standard of treatment. In the proper clinical scenario, a fasciotomy is indicated when compartment pressure exceeds 30 mm Hg [8]. If performed after 8 hours, however, there is often very little clinical benefit. Unfortunately, clinical outcomes for patients who undergo fasciotomy remain poor. Many often experience recurring neuropathic pain for many years.

## 4. Conclusion

Gluteal compartment syndrome is a rare condition that may develop after immobilization secondary to drug and alcohol abuse or improper surgical positioning. Delay in diagnosis can lead to nerve palsy, acute kidney injury, sepsis, and/or death. It is clinically relevant to make this diagnosis as early as possible in order to prevent unnecessary morbidity and mortality. Fasciotomy remains the gold standard therapeutic approach.
